# Biomass, carbon stock and sequestration potential of *Oxytenanthera abyssinica* forests in Lower Beles River Basin, Northwestern Ethiopia

**DOI:** 10.1186/s13021-021-00192-5

**Published:** 2021-09-17

**Authors:** Shiferaw Abebe, Amare Sewnet Minale, Demel Teketay, Durai Jayaraman, Trinh Thang Long

**Affiliations:** 1 Department of Geography and Environmental Studies, Bahir Dar University, P. O. Box 79, Bahir Dar, Ethiopia; 2 Department of Geography and Environmental Studies, Assosa University, P. O. Box 18, Assosa, Ethiopia; 3 Department of Range and Forest Resources, Botswana University of Agriculture and Natural Resources, Private Bag 0027, Gaborone, Botswana; 4 International Bamboo and Rattan Organization, Beijing, 100102 China

**Keywords:** Biomass, Carbon storage, Ethiopia, Lower Beles Basin, *O. abyssinica*, Soil carbon

## Abstract

**Background:**

Given the large bamboo resource base with considerable potential to act as an important carbon sink, Ethiopia has included bamboo in the national Reducing Emissions from Deforestation and Forest Degradation and enhancing forest carbon stocks (REDD+) and Clean Development Mechanisms (CDM) programs. However, little is known about the carbon stock and sequestration potential of bamboo forests. As a result, this research was conducted to quantify the carbon sequestration and storage capacity of *Oxytenanthera abyssinica* forests in the Lower Beles River Basin, northwestern Ethiopia. To this end, a total of 54 circular plots, each measuring 100 m^2^ with a radius of 5.64 m, were established to conduct the inventory in Assitsa and Eddida bamboo forests, the typical bamboo sites in Lower Beles River Basin. Biomass accumulation of bamboo was estimated using an allometric equation based on diameter at breast height (DBH) and age. Soil samples were taken from two different soil depths (0–15 and 15–30 cm) to determine soil organic carbon.

**Results:**

Results indicate that the mean biomass of the bamboo forests in the study area accounted for about 177.1 $\pm$ 3.1 Mg ha^−1^. The mean biomass carbon and soil organic carbon stock of the bamboo forests were 83.2 $\pm$ 1.5 Mg C ha^−1^ and 70 $\pm$ 1.7 Mg C ha^−1^, respectively. Therefore, the mean carbon stock of the *O. abyssinica* bamboo forests was 152.5 $\pm$ 2.5 Mg C ha^−1^ to 559.8 $\pm$ 9.0 ton CO_2_ ha^−1^.

**Conclusion:**

This study highlights the importance of assessing bamboo’s carbon stock and sequestration potential for enhancing its role in climate change mitigation and sustainable resource management. The *O. abyssinica* bamboo forests of the study area have significant carbon stock and sequestration potential. Therefore, sustainable management of these crucial vegetation resources will enhance their role in providing ecosystem services, including climate change mitigation.

## Background

In today’s world, climate change is one of the greatest challenges for humankind. Consequently, climate change mitigation has received the earnest attention of scientists, resource managers, and policymakers [1]. In this regard, carbon (C) sequestration by growing and managing forests has been recognized as the main mitigation strategy for the changing climate [1–3]. Forests are essential terrestrial C sinks because they store a large amount of C in vegetation and soil and interact with atmospheric processes through the absorption and respiration of CO_2_ [4–7]. Currently, forest resources, particularly tropical forests, are experiencing increasing pressure due to the growing population and land-use conversion resulting in deforestation and degradation [8]. Hence, the mounting rate of tropical deforestation makes the search for alternative natural resources critical. The characteristics of bamboo make it a perfect solution for the environmental and societal consequences of tropical deforestation [9].

Bamboo forests are an integral component of the tropical and sub-tropical forest ecosystems, covering 35 million hectares globally [10], and play an important role in climate change mitigation [11]. Plants sequester CO_2_ during photosynthesis and thus play an important role in climate regulation. However_,_ plants like bamboo have an extraordinary capacity to absorb CO_2_ from the atmosphere [12–14]. Bamboo is one of the fastest-growing plants [15], with growth rates ranging from 30 to 100 cm per day and a harvesting cycle of 3 to 5 years, compared to 10 to 50 years for most timber species [16–19]. On account of their fast growth and short harvesting cycle, bamboo forests have been known to have higher C storage per hectare than fast-growing tropical and sub-tropical trees under comparable conditions [7, 12, 18]. For example, Moso bamboo (*Phyllostachys edulis*) forest in China sequestered 5.10 Mg C ha^−1^ during a single year—a rate that is 33% higher than a tropical mountain rainforest and 41% higher than a 5-years old stand of *Cunninghamia lanceolata*, a fast-growing Chinese fir [20].

Indeed, bamboo has a lower total ecosystem C (94–392 Mg C ha^−1^) than timber forests (126–699 Mg C ha^−1^) [20, 21]. When C stored in harvested carbon-intensive bamboo products is taken into account, a managed giant bamboo species like Moso (*Phyllostachys Pubescens*) may store much higher carbon than a Chinese fir (296 versus 237 Mg C ha^−1^) growing under the same circumstances [22]. The management of bamboo has a significant effect on its C sequestration potential. For instance, appropriately managed (sufficient water, adequate nutrients, appropriate thinning, and regular harvesting) Moso bamboo sequester more C (296 Mg C ha^−1^) than bamboo in the natural state (49.5 Mg C ha^−1^) [21, 22]. In contrast, deforestation of bamboo forests could lead to significant C emissions, causing negative feedback to climate change [11].

Besides benefits for C accounting, bamboo provides many other ecosystem services. Bamboo is adaptive to adverse site conditions [23]; thus, it can be planted in degraded landscapes contributing significantly to the restoration of degraded lands, which are essential to combat desertification [24, 25]. With its extensive rhizome and root network and dense canopy, the presence of bamboo improves soil stabilization, increases slope stability, reduces soil erosion and run-off, and improves retention of nutrients and regulation of water flow in rivers and lakes [1, 12, 14, 26].

Given the potential for bamboo to act as an important C sink, there is a strong need to integrate bamboo into national and international policies and mechanisms aimed at managing the effects of global climate change [1, 27]. The most prominent international policy framework in this regard is the United Nations Framework Convention on Climate Change (UNFCCC), with its Clean Development Mechanism (CDM) and Reducing Emissions from Deforestation and Forest Degradation and enhancing forest carbon stocks (REDD+) [27]. Taking this view ahead, Ethiopia has amended its forest definition to include bamboo in the national REDD+ readiness and CDM projects.

Ethiopia set a vision to become a middle-income country with net-zero CO_2_ emission by 2030. In this regard, the implementation of REDD+ is expected to contribute 50% of the emission reduction target [28]. More profoundly, Ethiopia has prepared forest reference level (FRL) and submitted it to the United Nations Framework Convention on Climate Change (UNFCCC) in 2016 mainly to achieve result-based payment for verified emissions reductions [29]. Because of its suitable climate and environment for bamboo growth, Ethiopia possesses about 1.4 million hectares of bamboo resources [30]. Thus, it can be expected that Ethiopia plays a vital role in C sequestration and, thereby, climate change mitigation through bamboo forests. There is, therefore, a strong need to assess the C stock and sequestration potential of bamboo forests.

Globally, bamboo forests have received glutted attention for their high potential to sequester CO_2_ from the atmosphere. Several studies have been conducted on C stock and sequestration potentials of bamboo and demonstrated their potential as an efficient and effective C sink. Most of these studies were confined to Asia, mainly China [3, 7, 11, 14, 26, 31–34] and India [35–40], and focused on the common bamboo species, Moso bamboo [2, 7, 26, 31–34, 41]. Hence, further research is needed to reduce uncertainties in the underlying data, resulting from a lack of standardization of methods, a lack of research for many bamboo species, and limited research of belowground and soil organic carbon [20]. Another limitation identified by the same study is the under-representation of regions such as Central America, South America, and Africa, while most of the studies concentrated in Asia, mainly in China and India.

In Ethiopia, a few studies have been conducted on organic C stock and sequestration. However, most of these studies limited their scope to tree species while much less emphasis is given to the bamboo species. Given the large bamboo resource base on one hand, and the looming climate change on the other, information on C stock and sequestration potential of bamboo forests is critical for developing strategies that increase productivity (increase carbon stock) while reducing greenhouse gas emissions (enhancing carbon sequestration). In view of this backdrop, this study was conducted to estimate the biomass, C storage, and sequestration of *Oxytenanthera abyssinica* forests in the Lower Beles River Basin, north-western Ethiopia.

## Methods

### Description of the study area

**Fig. 1:**
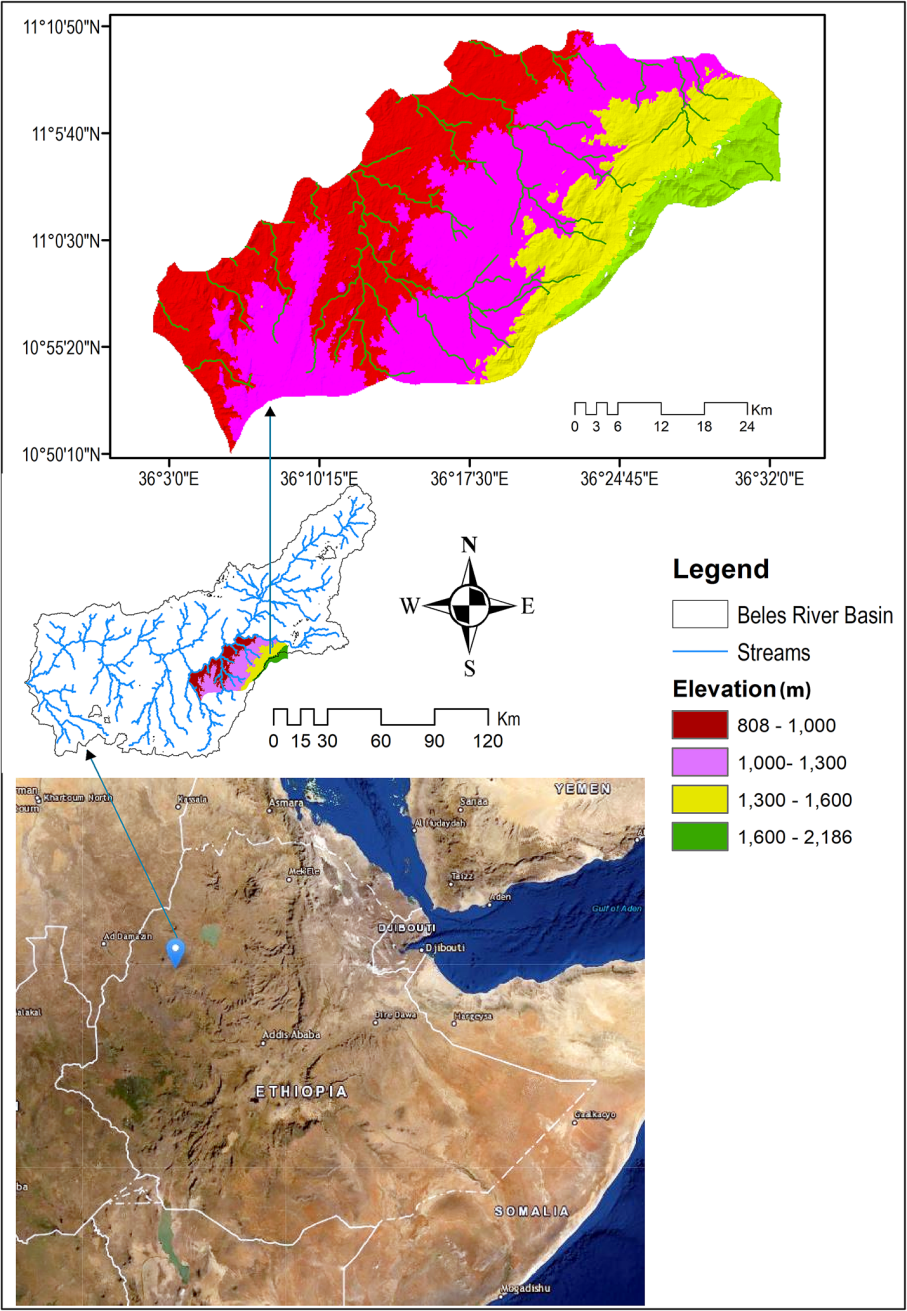
Map of the study area

This study was conducted in two *O. abyssinica* forests of Mandura District in the Lower Beles River Basin. The District is located between 10° 50′ 55ʺ to 11° 10′ 10ʺ N and 36° 02′ 48ʺ to 36° 32′ 42ʺ E in north-western Ethiopia (Fig. 1). It covers about 1045 km^2^ and is situated between 808 to 2186 m in north-western Ethiopia savannah lowlands. The largest portion of the District (lowland) is covered with basement complex rock when the plateau basalts cover few areas in the north-eastern part, specifically, the Kar Mountain escarpments [42]. The nitosols, cambisols, luvisols, and leptosols are the main soils of the sub-basin [43]. Temperatures range from a maximum of 35 to 40 °C to a minimum of 18 to 20 °C [44]. The rainfall shows a unimodal distribution and ranges approximately from 1300 to 2000 mm [43]. Generally, the District has a wet tropical climate (wet *kolla*) except few areas around the Kar Mountain, which is wet sub-tropical (wet *woynadega*).

The District has various types of vegetation resources that include *Boswellia papyrifera* (Del.) Hochst., *Anogeissus leiocarpa* (DC.) Guill. & Perr., *Stereospermum kunthianum* Cham. and the species of *Terminalia*, *Combretum,* and *Lannea*. The solid-stemmed Savanah or lowland bamboo [*Oxytenanthera abyssinica* (A. Rich.) Munro], locally known as *Shimel,* is one of the main vegetation types grown in the District [45]. It is a sympodial or pachymorphic rhizome bamboo species growing in the vast savannah lowlands of north-western Ethiopia [46] and takes the lion-share (85%) of bamboo resources of the country [47, 48]. Hence, two bamboo forest sites, namely, Assitsa and Eddida bamboo forests, were selected for the inventory based on their forest coverage and accessibility. These bamboo forests are under two separate management systems. The Assitsa bamboo forest is managed privately, while the Eddida forest is protected and managed by the state organ, the Ethiopian Biodiversity Institute. Bamboo grown in the Eddida forest is banned from harvesting by law.

### Sampling and data collection techniques

According to Huy and Long [49], if the forest investigation area is small (< about 20 ha) with discrete distribution, the application of random, systematic, or cluster layouts is not feasible and necessary; instead, typical sampling is applicable. In this case, 2–3 plots may be established in each forest block based on average density and bamboo size. Accordingly, a total of 54 circular plots, each measuring 100 m^2^ with a radius of 5.64 m, were established to conduct the inventory. Circular plots are more efficient because the actual perimeter of the plot is smaller than the square or rectangular plots; thus, the number of bamboo culms on the edge is limited [49].

In each plot, culms were classified based on their ages and, then, counted. A total of 30 culms per plot were randomly selected from each age group. Culm diameter was measured at 1.3 m height using a caliper, and a graduated stick (graduated bamboo culm marked at 1 m intervals) was used to measure the height of culms. The culm age was determined based on the exterior color of the culm, features of the culm sheath, and the development of branches and leaves [50]. Hence, culm ages were determined as follows: (a) 1-year-old bamboo culms are those that emerged in the current year and have only a few leaves, the sheath on the culm, and the culm has pale surface color covered with a white powder; (b) 2-years-old bamboo culms have few sheaths at the base of culm with some beginning to rot, well-developed branches on 5th and 6th internodes, and the white powder on the culm surface also beginning to disappear while the culm is turning dark green; (c) 3-years-old bamboo culms have no sheaths, and the culm bottom turned dark green, symbolizing near maturity, and characterized by the appearance of few lichens and mosses on culm surfaces; and (d) 4-years-old culms have no sheath, and the culm color is light yellowish green with an abundance of lichens and mosses, and (e) 5-years-old or older bamboo culms have the culm surface covered with an abundance of mosses and lichens and had turned brownish green in color [18, 50].

Soil carbon is likely to vary with depth. As a result, most soil carbon samplings define one or more soil layers, usually by the distance in centimeters from the soil surface [51]. According to IPCC [52], 30 cm is suggested as the default sampling depth for forest soil to determine carbon content. Lowland bamboo is a shallow-rooted plant where the average depth of dense roots reaches 30 cm [53]. As a result, it makes sense to define the top layer as 0–30 cm and subdivide it further into 0–10 and 10–30 cm layers [54]. Accordingly, in this study, a total of 108 (54 plots × 2 depths) soil samples were taken from two different soil depths: 0–15 cm and 15–30 cm for determining the soil texture, moisture, pH, concentrations of organic carbon (OC), and total nitrogen (TN). The soil samples collected from each depth were composited separately, labeled, and placed in durable plastic bags. Similarly, a total of 108 (54 plots × 2 depths) undisturbed soil samples were taken at the middle of each plot using a soil core sampler to estimate soil bulk density.

### Soil laboratory analyses

Soil samples were analyzed in the Agricultural Research Laboratory of the Ethiopian Institute of Agricultural Research in Pawi, Ethiopia. Before conducting the chemical analyses, soil samples were air-dried and pounded with pestle and mortar, and passed through a 2 mm sieve. Then, the soil moisture content was determined by the percentage loss of soil sample after it was oven-dried at 105 °C divided by the dried soil weight in gram [55], while soil texture was determined through the hydrometer method [56]. The bulk density analysis was carried out using the core sampling method [57], where each sample was dried at 105 °C for a minimum of 48 h. The volume of the core sampler ($\mathrm{VC}$) was determined by using the formula: $\mathrm{VC}=\uppi {r}^{2}\mathrm{h},$ where r is the radius and h is the height of the core sampler [$\mathrm{VC}=3.14 \times {(2.5\,\mathrm{cm})}^{2} \times 5\,\mathrm{cm}=98.125\,{\mathrm{cm}}^{3}$]. Soil pH was determined in 1:2.5 pH-H_2_O ration using the Beckman Zeromatic-II pH meter. The Walkley and Black [58] and micro-Kjeldahl methods [59] were used to determine SOC and TN concentrations, respectively.

### Data analyses

#### Estimation of biomass, carbon stocks and CO_2_ sequestration

The aboveground biomass (AGB) storage of *O. abyssinica* forests was estimated using an allometric model developed by Amoah et al. [18]. The model was developed by harvesting randomly selected 1–2, 3–4, and 5–6 year-old culms of *O. abyssinica* in Ghana. Five culms per age group were harvested; thus, a total of 15 culms were harvested from five plots (10 × 10 m) based on age and diameter. The mean DBH of the sampled culms was 1.92 (1–2 year), 1.84 (3–4 year), and 1.1 for 5–6 year-old culms. The model has a robust predictive power with R^2^ in the range between 0.868–0.916. In this model, the relationship between AGB and DBH were predicted by a simple allometric function:

$$\mathrm{Y}=a \times {\mathrm{DBH}}^{b}$$

where Y = aboveground biomass and $a$ and $b$ are parameters.

$${\mathrm{AGB}}_{1} \left(1-2\;\mathrm{years}\right)=2.632 \times {\mathrm{DBH}}^{1.881}$$

$${\mathrm{AGB}}_{2} \left(3-4\;\mathrm{ years}\right)=1.910 \times {\mathrm{DBH}}^{2.410}$$

$${\mathrm{AGB}}_{3} \left(5-6\;\mathrm{ years}\right)=2.304 \times {\mathrm{DBH}}^{2.233}$$

Then, the total aboveground biomass (TAGB) was calculated by summing up of aboveground biomass of each age class as follows

$$\mathrm{TAGB}={\mathrm{AGB}}_{1}+{\mathrm{AGB}}_{2}+{\mathrm{AGB}}_{3}$$

*Oxytenathera abyssinica* species has pachymorphic rhizome, hence invest more resources to belowground parts for its structural stability. As a result, is stores higher amount of belowground biomass. Hence, the high AGB to belowground biomass (BGB) ratio of 1:4 was used to estimate the BGB [60]. The BGB was then computed as follows:

$$\mathrm{BGB}=\mathrm{AGB} \times 0.25.$$

Then, total belowground biomass $(\mathrm{TBGB})={\mathrm{BGB}}_{1}+ {\mathrm{BGB}}_{2}+{\mathrm{BGB}}_{3}$. Finally, C storage and CO_2_ equivalent potential of the bamboo forest was calculated from total biomass (TB) (total aboveground and belowground), respectively, as follows [52]:

$$\mathrm{Carbon }\left(\mathrm{C}\right)=\mathrm{C fraction }\left(0.47\right)\times \mathrm{TB}; \mathrm{and}\;{\mathrm{CO}}_{2}=\mathrm{C} \times 3.67$$

### Soil carbon and nitrogen stock

The amount of carbon and nitrogen stored per hectare was derived from soil depth (cm), bulk density (g cm^3^), and the percentage of soil organic carbon (SOC) and total nitrogen (TN) based on the following formula [61]: $$\mathrm{SOC\; stock}=\mathrm{BD}*\mathrm{d}*\% \mathrm{SOC};\mathrm{and\; TN\; stock}=\mathrm{BD}*\mathrm{d}*\% \mathrm{TN}$$ where SOC stock = Soil organic carbon stock per unit area (Mg C ha^−1^), TN stocks = total nitrogen stocks (Mg N ha^−1^), BD = soil bulk density (g cm^3^), d = depth of the sampled soil layer (cm), % SOC = percentage organic carbon and TN (%) = total nitrogen concentration. Then, the CO_2_ sequestration potential of bamboo forest soil was calculated by multiplying C with 3.67 [52]. Bulk density (BD) was calculated from the following formula [61]

$$\mathrm{BD}=\frac{\mathrm{MS}}{\mathrm{VC}},$$

where BD = Bulk density (g cm^3^); MS = Mass of the dried soil (g); VC = volume of core sampler (cm^3^).

### Statistical analysis

A pairwise comparison was conducted to assess the mean difference of bamboo forests and soil depth depending on soil properties. Also, multiple mean comparisons were employed for each variable (texture, soil moisture, pH, bulk density, nitrogen content, organic carbon content, soil organic carbon, and nitrogen stock) at $\mathrm{\alpha }=0.05$ using the DUNCAN test. Correlation analysis was carried out to test the association between soil bulk density and SOC along with soil depth.

## Results

### Culm density and stand structure of *O. abyssinica* forests

The culm density of *O. abyssinica* stand showed significant variation between the study sites, Assitsa (12,364) and Eddida (15,025) bamboo forests. In terms of culm age composition, 3–4 year-old bamboo culms were the most common (5921 culms ha^−1^ = 47.9%) in the stand, while 5–6 and 1–2 year-old culms made up 26.1% (3236 culms ha^−1^) and 25.9% (3207 culms ha^−1^) of the culms in Assitsa forest, respectively. 5–6-year-old culms (6035 culms ha^−1^ = 40.2%) and 3–4-year-old culms (5505 culms ha^−1^ = 36.6%) made up 76.8% of the bamboo stand in Eddida bamboo forest, while 1–2-year-old culms shared the remaining proportion (3485 culms ha^−1^ = 23.2%) (Table 1).

**Table 1:** Stand structure of *O. abyssinica* forests in the study area

Age (year)	Assitsa forest				Eddida forest			
	Culm density (ha^−1^)	Clump density (ha^−1^)	Mean DBH (cm)	Mean height (m)	Culm density (ha^−1^)	Clump density (ha^−1^)	Mean DBH (cm)	Mean height (m)
1–2	3207		4 $\pm$ 0.41	11.4 $\pm \, 0.12$	3485		4.5 $\pm\, 0.27$	12.2 $\pm\, 0.47$
3–4	5921		3.8 $\pm$ 0.42	11.0 $\pm$ 0.12	5505		4.1 $\pm \,0.27$	11.9 $\pm \, 0.52$
5–6	3236		3.5 $\pm \,0.4$	10.8 $\pm \,0.12$	6035		3.8 $\pm \,0.23$	11.8 $\pm \,0.53$
Total	12364	671	3.8 $\pm \,0.41$	11 $\pm \,0.12$	15025	518	4.1 $\pm \,0.25$	11.96 $\pm \,0.50$

**Fig. 2:**
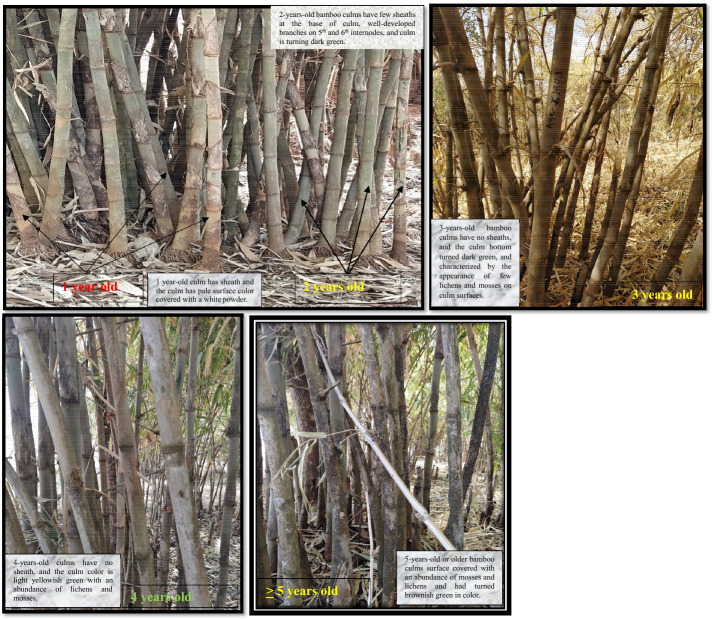
Field photos showing *O. abyssinica* bamboo culm age in the study area

The 1–2 year-old culms have the highest thickness (DBH) both in Assitsa (4 $\pm$ 0.41 cm) and Eddida (4.5 cm $\pm \,0.27$) bamboo forests. In contrast, 5–6 year-old culms had the lowest DBH value of 3.5 $\pm \,0.4$ and 3.8 cm$\pm \,0.23$ for Assitsa and Eddida bamboo forests, respectively. As age increases, the thickness of bamboo culm decreases (Table 1, Fig. 2).

### Biomass and carbon storage potential of *O. abyssinica* forests

Results indicate a significant variation in the total biomass storage (AGB and BGB) between the bamboo forests of the study area (Table 2). The mean biomass was higher in Eddida (185.1 $\pm$ 3.1 Mg ha^−1^) than Assista (154.3 $\pm$ 5.2 Mg ha^−1^) bamboo forests. The mean total biomass and biomass C stock of the studied bamboo forests were 177.1 $\pm$ 3.1 Mg ha^−1^ and 83.2 Mg$\pm \,1.5$ C ha^−1^, respectively.

**Table 2:** Biomass and carbon storage potential of *O. abyssinica* forests

Bamboo forest	AGB (Mg ha^−1^)	BGB (Mg ha^−1^)	TB (Mg ha^−1^)	CS (Mg C ha^−1^)
Assitsa	123.47 $\pm \,4.16$	30.86 $\pm$ 1.04	154.34 $\pm \,5.20$	72.54 $\pm \,2.44$
Eddida	148.07 $\pm \,2.46$	37.01 $\pm \,0.61$	185.10 $\pm \,3.077$	86.99 $\pm \,1.44$
Mean $\pm \,\mathrm{SE}$	141.69 $\pm$ 2.56	35.42 $\pm$ 0.64	177.12 $\pm \,3.21$	83.24 $\pm \,1.50$

*AGB* above ground biomass, *BGB* below ground biomass, *TB* total biomass, *CS* carbon stock

### Physicochemical properties of soils of *O. abyssinica* forests

The sand fraction was the dominant (63.3 $\pm$ 0.1%) textural class, while the silt textural class shared the smallest (14.2 $\pm$ 0.5%) proportion on both topsoil (0–15 cm) and subsoil (15–30 cm). SOC and N content decreased, while BD and moisture content increased along with soil depth. Likewise, soil _P_H decreased as soil depth increased. The SOC concentration decreased from 5.3 to 2.8% in the Eddida forest and 3.4 to 2.4% in the Assitsa bamboo forests for 0–15 to 15–30 cm soil depths, respectively. TN concentration on the topsoil to sub-soil layer decreased from 0.38 to 0.21 and 0.25 to 0.18% in the Eddida and Assitsa bamboo forests, respectively. The analysis indicates that SOC and TN concentration varied significantly (P < 0.05) across soil depth (Table 3).

**Table 3:** Soil physicochemical properties and carbon stocks of *O. abyssinica* forests

Parameters	Soil depth (cm)	Bamboo forests	
		Assitsa	Eddida
Clay, silt and sand (%)	0–15	20:10:70	18:16:66
	15–30	22:12:66	28:18:54
Soil _P_H	0–15	5.99 $\pm \,0.55$	5.86 $\pm \,0.80$
	15–30	5.84 $\pm \,0.36$	5.58 $\pm \,0.35$
SOC (%)	0–15	3.4 $\pm \,0.19$	5.3^a^ $\pm \,0.17$
	15–30	2.4 $\pm \,0.14$	2.8^b^ $\pm \,0.14$
TN (%)	0–15	0.25 $\pm \,0.01$	0.38^a^ $\pm \,0.12$
	15–30	0.18 $\pm \,0.10$	0.21^b^ $\pm \,0.00$
BD (g cm^−3^)	0–15	1.34^a^ $\pm \,0.39$	1.14^a^ $\pm \,0.27$
	15–30	1.55^b^ $\pm \,0.27$	1.30^b^ $\pm \,0.27$
Moisture (%)	0–15	8.23 $\pm \,0.56$	9.69^a^ $\pm \,0.31$
	15–30	9.60 $\pm \,1.00$	12.57^b^ $\pm \,0.72$
TN stock (Mg N ha^−1^)	0–15	5.04 $\pm \,0.17$	6.49^a^ $\pm \,0.22$
	15–30	4.36 $\pm \,0.22$	4.18^b^ $\pm \,0.19$
SOC stock (Mg C ha^−1^)	0–15	67.40 $\pm \,2.60$	90.25^a^ $\pm \,3.62$
	15–30	56.07 $\pm \,3.32$	54.08^b^ $\pm \,2.66$

Values are means and standard errors (Mean ± SE). Letters along columns denote significant difference at P < 0.05

*SOC* soil organic carbon, *TN* total nitrogen, *BD* soil bulk density

**Fig. 3:**
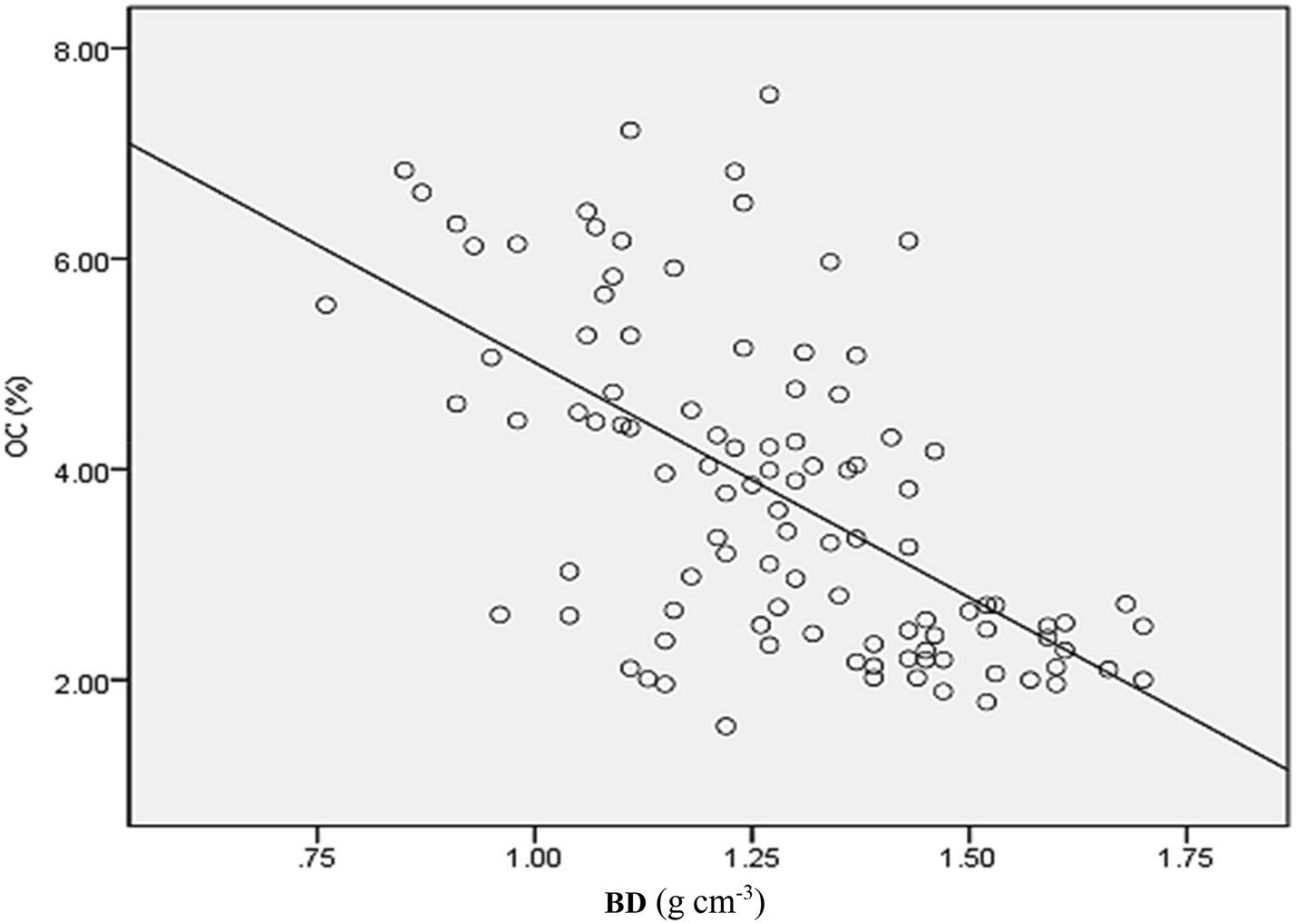
The relationship between organic carbon (OC) and bulk density (BD)

While associating BD and SOC content, a strong correlation (r = − 0.7) was found in the study. The result indicated that as BD increases, OC decreases, and vice-versa (Fig. 3). SOC concentration decreased from 4.8 on the topsoil to 2.6% on the sub-soil layer, while BD increased from 1.19 to 1.36 g cm^−3^ on the topsoil to subsurface soil. Soil BD was significantly different (P < 0.05) across soil depths. Soil _P_H values decreased on the topsoil to sub-soil from 5.88 to 5.66. Conversely, soil moisture increased from 9.57 (0–15 cm) to 11.13% in 15–30 cm soil depth. The lower moisture content in the topsoil could be due to the surface soil exposure to solar radiation and disturbances, which would lead to loss of soil moisture through the evapotranspiration process.

### Total carbon stocks and sequestration potential of *O. abyssinica* forests

Mean biomass (AGB and BGB) C and SOC stocks (0–30 cm) were 72.5 $\pm \,2.4$ and 61.7 Mg $\pm \,2$ C ha^−1^ for Assitsa bamboo forest, respectively, and 87 $\pm \,1.4$ and 72 Mg $\pm \,2.0$ C ha^−1^ for Eddida bamboo forest, respectively (Table 4). The mean total biomass C and SOC stock of the two forests was 159.5 $\pm \,3.9$ and 133.7 Mg $\pm \,4$ C ha^−1^, respectively. Therefore, the mean total C stock of the *O. abyssinica* forests in February 2020 was 293.2 $\pm$ 8 Mg C ha^−1^ with 1076 $\pm$ 20.2 ton CO_2_ ha^−1^.

**Table 4:** Total carbon stocks and CO_2_ sequestration of *O. abyssinica* forests

Bamboo	Carbon pools				
Forest	AGB (Mg C ha^−1^)	BGB (Mg C ha^−1^)	SOC (Mg C ha^−1^)	TCS (Mg C ha^−1^)	CO_2_ Seq. (ton ha^−1^)
Assitsa	58.03 $\pm \,1.95$	14.50 $\pm \,0.48$	61.73 $\pm \,1.94$	134.27 $\pm \,3.07$	492.80 $\pm \,11.28$
Eddida	69.59 $\pm \,1.15$	17.39 $\pm \,0.28$	72.57 $\pm \,2.23$	158.91 $\pm \,2.44$	583.22 $\pm \,8.96$
Mean $\pm \,\mathrm{SE}$	66.59 $\pm \,1.20$	16.64 $\pm \,0.30$	69.70 $\pm \,1.83$	152.52 $\pm \,2.45$	559.78 $\pm \,9.02$

*AGB* above ground biomass, *BGB* below ground biomass, *SOC* soil organic carbon (0–30 cm), *TCS* total carbon stock, *CO*_*2*_* seq.* CO_2_ sequestration

## Discussion

### Culm density and stand structure of *O. abyssinica* forests

Results indicate that *O*. *abyssinica* forests of the present study area have significant variations in both clump and culm density. The Eddida bamboo forest had a higher (15,025 culms ha^−1^) culm density than the Assitsa bamboo forest (12,364). Conversely, clump density was higher in the Assista bamboo forests (671 ha^−1^) than Eddida bamboo forest (518 ha^−1^). The harvesting intensity and season play a significant role in determining the overall culm density of the studied bamboo forests. Several harvested culms were observed, where some clumps had fewer culms in the Assitsa bamboo forest during the field survey and inventory. In contrast, in Eddida bamboo forest, where bamboo harvesting is only allowed for research purposes, most bamboo culms have been in their natural stand since the bamboo forest reserve establishment.

Compared to the present study, a much lower density of 4374 culms ha^−1^ was recorded for *O. abyssinica* from Cameron [62]. Densities of 6267 culms ha^−1^ was reported for the same bamboo species from Ghana [18], while 8840 culms ha^−1^ was reported for highland bamboo (*Yushania alpina*) from Ethiopia [63]. Comparable densities of 20,467 culms ha^−1^ were reported by Mulatu and Fetene [64] and 20,748 culms ha^−1^ [65] for highland bamboo (*Yushania alpina*) in Ethiopia. Generally, bamboo stand density depends on plant species, tending operations, harvesting intensity, and site conditions [18, 32, 50].

Moreover, this study indicated that the youngest (1–2 year old) culm has the highest DBH (4.25 $\pm$ 0.34). As the age of bamboo culm increases, DBH tends to decrease. Unlike trees, bamboo have no secondary meristem; the cambium, thus, lacks special tissues to accumulate or shade metabolic residues [66, 67]. Consequently, the existing bamboo tissues have to function for many years without forming any new tissues [68]. The aging of bamboo culms is associated with significant structural and chemical changes in the parenchyma and fiber tissues, including cell wall thickening, declining moisture content, and an increase or decrease in certain nutrient ions [68, 69].

### Biomass and carbon storage potential of *O. abyssinica* forests

The results revealed that total biomass stored by bamboo forests was higher in the Eddida (185.1 $\pm$ 3.1 Mg ha^−1^) than in the Assista bamboo forest (154.3 $\pm$ 5.2 Mg ha^−1^). The studied bamboo forests stored total mean biomass of 177.1 $\pm$ 3.2 Mg ha^−1^ in the AGB (141.7 $\pm \,2.6$) and BGB (35.4 $\pm \,0.6$). The AGB stored by bamboo forests of the present study sites is much greater than the corresponding reported values of 28 Mg ha^−1^ AGB for *O. abyssinica* from Cameron [62]*,* and 100 Mg ha^−1^ of AGB from Kenya [70] and 110.7 Mg ha^−1^ AGB from Ethiopia for *Yushinia alpina* [63]; 99 Mg ha^−1^ [64] and 108.7 Mg ha^−1^ [65] for total biomass of *Y. alpina* in Ethiopia*.* Likewise*,* 114 Mg ha^−1^ AGB was reported for *B. vulgaris*, while significantly lower values of 4.2 Mg ha^−1^ were recorded for *O. abyssinica* in Ghana [18]. Generally, the results indicated that bamboo stand biomass accumulation depends upon culm size (diameter), species, density, and management practice.

The present study found that the total organic carbon stored by bamboo forests varied between the study sites. The total biomass C stored by bamboo forests was higher in the Eddida (87 $\pm$ 1.4 Mg C ha^−1^) than in Assitsa bamboo forest (72.5 $\pm$ 2.4 Mg C ha^−1^). The aboveground carbon stored by the studied bamboo forests (58.0 $\pm$ 1.6–69.6 $\pm$ 1.2 Mg C ha^−1^) is much greater than the values reported for the same species, in Ghana (2 Mg C ha^−1^) [18] and Cameron (13.1 Mg C ha^−1^) [62]. Similarly, lower values of 33.9 Mg C ha^−1^ for *B. vulgaris var vitata* and 50.8 Mg C ha^−1^ for *B. vulgaris* from Ghana [18]. The total biomass C stored by the studied bamboo is considerably higher than 40–43.5 Mg C ha^−1^ for *Y. alpina* were reported from Ethiopia [71]. Compared to timber plant species, C stored by the studied bamboo forests biomass is much higher than a value of 22.3 Mg C ha^−1^ stored by herbivore exclosures, and 58.1 Mg C ha^−1^ for managed natural dry forests of north Ethiopia [72]. Generally, C storage of bamboo forests biomass depends on species, management practices, and site conditions [32, 50].

### Soil organic carbon content of *O. abyssinica* forests

Soils of the study landscape are slightly acidic, and sandy clay loam in texture. Soil pH ranged between 5.9 and 5.7 in the topsoil and sub-surface soil layers, respectively. Soil bulk density and moisture content increased with increasing soil depth. Conversely, the quantity of C and N stored in the bamboo forest soil decreased along with increasing soil depth. This indicates that organic matter concentration is higher at the topsoil (0–15 cm) than at the sub-surface soil layer. In this regard, similar studies conducted so far confirmed that SOC decreased while bulk density increased with increasing depth in bamboo forest soils [1, 12, 33, 73, 74].

SOC storage (0–30 cm) ranged from 61.7 to 72 Mg C ha^−1^ with a mean of 70 $\pm$ 1.7 Mg C ha^−1^ for bamboo forests of the study area. C stored in the bamboo forest soils (70 Mg C ha^−1^) is lower than C stored in the bamboo forest biomass (83.2 $\pm$ 1.5 C Mg ha^−1^). Similar to the present study, vegetation biomass has been reported to accumulate higher carbon than forest soils in Ethiopia [75, 76]. A meta-study by Yuen et al. [20] based on 184 bamboo carbon studies review confirms that the SOC in bamboo forests ranges from 70 to 200 Mg C ha^−1^. In general, the results indicated that bamboo forest soils have an important role in the sequestration of atmospheric CO_2,_ as do the soils of tropical forests.

### Total carbon stocks and sequestration potential of *O. abyssinica* forests

The total carbon stock of the studied bamboo forests (134.3 $\pm$ 3.1–159 $\pm$ 2.4 Mg C ha^−1^) is within the range of values 94–392 Mg C ha^−1^, estimated by Yuen et al. [20] for total C stored in bamboo plant biomass and soil (bamboo ecosystem). Compared to other forest species, C stored in the bamboo ecosystem of the studied forests is lower than the reported values of 340.9 Mg C ha^−1^ for dry Afromontane forests of north-western Ethiopia [76], 400 Mg C ha^−1^ for Yegof mountain natural vegetation of north-east Ethiopia [77], and 354.5 Mg C ha^−1^ for *Eucalyptus Saligna* plantations in central highlands of Ethiopia [78], and 496.3 and 277.4 Mg C ha^−1^ were reported for natural forest and coffee agroforestry, respectively, from eastern Ethiopia [79]. Likewise, our C estimate is lower than C estimates of 337 and 274 Mg C ha^−1^ for primary and secondary tropical forests of Singapore [80]. However, our result was higher than the reported values of 77–135 Mg C ha^−1^ for diversified woody-based dryland agroforestry systems in northern Ethiopia [81]. The total C stock of the studied bamboo forests was comparable with the values of 134.1 Mg C ha^−1^ for *Schizostachyum pergracile* bamboo forest of Manipur northeast India [12], and 145.4 Mg C ha^−1^ Moso bamboo (*Phyllostachys pubescens Mazel ex Houz.*) from southern China [74].

By and large, this study signifies the reporting and verification of Ethiopia’s performance in terms of emissions reduction or carbon removal via its bamboo resources. In addition, a considerable amount of revenue can be generated through selling carbon credits in the carbon market via the Clean Development Mechanism (CDM) projects that will enhance the socio-economic development of Ethiopia.

Due to resource constraints, we were unable to develop an allometric model for estimating both aboveground and belowground biomass in this study. We used a published allometric equation developed by Amoah et al. [18] to estimate AGB. The model was indeed developed from the same species (*O. abyssinica*), but it may not accurately estimate the AGB as the site conditions vary from place to place. At the same time, BGB was calculated based on the root to shoot ratio method provided by Singar et al. [60]. This may underestimate or overestimate the BGB stored by the studied bamboo forests. Therefore, a local or site and species-specific model has to be developed to determine the biomass and C storage capacity of lowland bamboo forests with better precision.

### Enhancement of bamboo stands to maximize biomass carbon storage

It is estimated that about 35 billion tons of CO_2_ equivalent is produced each year by human activities [82]. By virtue of its fast growth, bamboo offers one of the quickest ways to sequester vast amounts of that CO_2_ from the atmosphere [83, 84]. Several studies have evidenced that the CO_2_ sequestration potential of bamboo equals or surpasses that of fast-growing trees over short periods [12, 24, 27, 31, 84]. Indeed, studies have reported that regularly managed Moso bamboo accumulates higher biomass and, hence, more carbon by a factor of 2.39 than fast-growing tree species, such as Chinese fir [31, 83, 85].

Bamboo forests of the study area can accumulate 339.4 Mg ha^−1^ biomass and store 293.2 Mg ha^−1^ of C, and sequester 1076 tons of CO_2_ ha^−1^. If the bamboo forests had been properly managed, they could have stored more carbon. Furthermore, bamboo could provide an important and long-term C sink if harvested bamboo culms are turned into durable products such as permanent construction materials, furniture, art, etc. [22, 50]. Due to recent technological developments, bamboo is being used to produce various durable products that can last for 30 years or more [22]. Over 30 years, a significant amount of carbon can be locked into durable bamboo products. Thus, managing bamboo forests will enhance their capacity to store C in its ecosystem and products.

During field survey and inventory, it was observed that bamboo forests were not managed. In Assitsa forest, bamboo was harvested and managed traditionally by farmers based on the lesson they learnt from their fathers and forefathers and personal experiences. Their knowledge, however, is insufficient to appropriately manage bamboo forests. In this regard, an earlier study by Kelbessa et al. [46] reported that the farmers and communities have a relatively low level of knowledge and skills to manage bamboo forests. In the state-owned Eddida forest, bamboo harvesting is prohibited except for research purposes. Hence, most bamboo culms are in their natural state, where the majority (76.3%) are old (5–6 year old) and matured (3–4 year old) culms, while young culms share the remaining proportion of 23.7% of the stand. In this respect, harvesting of matured and old culms has been recommended to stimulate the growth of rhizomes and roots, increase shoot production in a clump, and have positive carbon sequestration [4, 18, 27, 50]. However, harvested culms should be turned into durable products (such as bamboo furniture, flooring, and houses) for effective long-term sequestration of carbon [20, 83].

Moreover, in the studied bamboo forests, it was observed that improper harvesting and management practices occurred extensively, such as harvesting of young and immature culms, cutting of bamboo culms at the higher position from root base, and unseasonal harvesting mainly, during wet seasons. Nevertheless, only matured culms of age three and above are recommended for harvest [86]. Furthermore, bamboo should be harvested during the dry season, when the culm’s moisture content is low and new shoots, which mostly emerge during the wet season, are not damaged. Moreover, cutting bamboo culms at higher positions does not relieve bamboo stands from clump congestion; rather, it hinders the emergence and growth of new shoots [69].

Generally, adopting scientific harvesting and management techniques plays a crucial role in enhancing the biomass productivity of bamboo stands and carbon sequestration. Moreover, improved management practices such as weeding, soil loosening, selective thinning of old and malformed culms, and nutrient management increase the productivity of bamboo stands. In addition, protecting bamboo stands from disturbances such as wildfire and animal interferences for grazing is reported to increase culm yield by 158–519% [69].

## Conclusions

This study confirms the critical role of lowland bamboo forests in C sequestration and, thus, climate change mitigation. The lowland bamboo forests of the study area can accumulate 339.4 Mg ha^−1^ biomass and store 127.6, 31.9, and 133.7 Mg C ha^−1^ in its AGB, BGB, and soil, respectively. Hence, these forests could store a total of 293.2 Mg C ha^−1^ and sequester 1076 ton CO_2_ ha^−1^ in their ecosystems. C stored in the studied bamboo forests is lower than in dry Afromontane forests (340.9 Mg C ha^−1^) of north-western Ethiopia [76], *Eucalyptus Saligna* plantation (354.5 Mg C ha^−1^) of central highlands of Ethiopia [78], and natural forest (496.3 Mg C ha^−1^) and coffee agroforestry (277.4 Mg C ha^−1^) of eastern Ethiopia [79]. Our result, however, was higher than the previously reported values of 77–135 Mg C ha^−1^ for diversified woody-based dryland agroforestry systems in northern Ethiopia [81]. The total C stock of the studied bamboo forests was comparable to that of the *Schizostachyum pergracile* bamboo forest (134.1 Mg C ha^−1^) of Manipur, northeast India [12] and the Moso bamboo (*Phyllostachys pubescens Mazel ex Houz*.) of southern China (145.4 Mg C ha^−1^) [74].

If the bamboo forests are properly managed and harvested culms are turned into durable products, they could sequester more C and provide an essential and long-term C sink. However, bamboo have been managed rudimentarily, hindering their sustainability and role in sequestering more C and providing other ecosystem services. Therefore, we strongly recommend the sustainable management of bamboo forests to enhance their productivity and function in providing ecosystem services, including climate change mitigation.
